# Case report: Structural brain abnormalities in *TUBA1A*-tubulinopathies: a narrative review

**DOI:** 10.3389/fped.2023.1210272

**Published:** 2023-09-08

**Authors:** Piero Pavone, Pasquale Striano, Giovanni Cacciaguerra, Simona Domenica Marino, Enrico Parano, Xena Giada Pappalardo, Raffaele Falsaperla, Martino Ruggieri

**Affiliations:** ^1^ Section of Pediatrics and Child Neuropsychiatry, Department of Child and Experimental Medicine, University of Catania, Catania, Italy; ^2^ National Council of Research, Institute for Biomedical Research and Innovation (IRIB), Unit of Catania, Catania, Italy; ^3^ Department of Neurosciences, Rehabilitation, Ophthalmology, Genetics, Maternal and Child Health, University of Genoa, Genova, Italy; ^4^ Pediatric Neurology and Muscular Diseases Unit, IRCCS Istituto “G. Gaslini”, Genova, Italy; ^5^ Pediatrics and Pediatric Emergency Department, University Hospital, A.U.O “Policlinico-Vittorio Emanuele”, Catania, Italy

**Keywords:** *TUBA1A*-tubulinopathy, cerebral anomalies, Dandy–Walker Malformation, DWM phenotype, tubulinopathies

## Abstract

**Introduction:**

Tubulin genes have been related to severe neurological complications and the term “tubulinopathy” now refers to a heterogeneous group of disorders involving an extensive family of tubulin genes with *TUBA1A* being the most common. A review was carried out on the complex and severe brain abnormalities associated with this genetic anomaly.

**Methods:**

A literature review of the cases of *TUBA1A*-tubulopathy was performed to investigate the molecular findings linked with cerebral anomalies and to describe the clinical and neuroradiological features related to this genetic disorder.

**Results:**

Clinical manifestations of *TUBA1A*-tubulinopathy patients are heterogeneous and severe ranging from craniofacial dysmorphism, notable developmental delay, and intellectual delay to early-onset seizures, neuroradiologically associated with complex abnormalities. *TUBA1A*-tubulinopathy may display various and complex cortical and subcortical malformations.

**Discussion:**

A range of clinical manifestations related to different cerebral structures involved may be observed in patients with *TUBA1A*-tubulinopathy. Genotype–phenotype correlations are discussed here. Individuals with cortical and subcortical anomalies should be screened also for pathogenic variants in *TUBA1A*.

## Introduction

The term “tubulinopathy” indicates a heterogeneous group of disorders involving tubulin genes and presenting with complex cerebral malformations and severe neurological manifestations (1–7). Tubulin genes are an extensive family of genes composed of alpha, beta, gamma, delta, and epsilon. The tubulin alpha includes about 15 genes and the tubulin beta about 23 genes, respectively. Tubulin alpha and tubulin beta encode tubulin proteins that form heterodimers that are fundamental components of microtubules. Microtubules play an important role in brain developmental processes including mitosis, neuronal migration, synaptic connectivity, and axonal transport (3–9). Dysfunction of microtubule-dependent functions from variants in components alpha, beta, and gamma cause severe cerebral malformations and neurological clinical involvement (10). The tubulin genes mainly involved in cerebral malformations are *TUBA1A* (MIM#602529), *TUBA8* (MIM#605742), *TUBB2A* (MIM#615101), *TUBB2B* (MIM#612850), *TUBB3* (MIM#602661), *TUBB5* (MIM#191130), and *TUBG1* (MIM#191135) (3–5). The different tubulin isotypes have sequence homology with diverse spatiotemporal expressions prompting a unique, isotype-specific function (10, 11). Among the patients affected by tubulinopathies, the most common mutation is represented by *TUBA1A*, coding the alpha-1 tubulin that is selectively and mainly expressed in post-mitotic neurons. Keays et al. (1) first reported mutations in alpha-tubulin as a cause of abnormal neuronal migration in mice and of lissencephaly (LIS) in humans. *TUBA1A* (alpha-tubulin complex) gene is located in chromosome 12q12-q14 and is composed of the N-terminal, intermediate, and C-terminal domains (12, 13). According to Hebebrand et al. (5), the variants in the affected individuals are shown to be distributed in clustering mainly around the Arg 402 residue in exon 4 in the C-terminal domain. Alpha-tubulinopathy manifests with a more severe phenotype than beta-tubulinopathy and accounts for 4%–5% of all the reported cases of lissencephaly (2, 4, 5). The clinical expression of patients affected by *TUBA1A*-tubulinopathy is various and complex such as craniofacial dysmorphism, severe developmental delay, cerebral palsy, and early-onset epilepsy. Cortical malformations such as lissencephaly, polymicrogyria (PMG), cortical gyral simplification, and gray matter heterotopias are frequently reported as well as anomalies of the corpus callosum, cerebellar vermis, cerebellum, and basal ganglia. Ventricular dilatation, anomalies of brainstem, abnormal hippocampus, and internal capsule are also reported (7, 13–19).

We reviewed the reported cases of *TUBA1A*-tubulinopathy underlying the frequent and severe neurological involvement associated with this genetic abnormality.

## Material and methods

### CARE checklist and review search strategy

This study was conducted according to the CARE guidelines (www.care-statement.org/checklist). A literature review related to the area of focus was conducted by collecting clinical trials, primary research, and reviews from online bibliographic databases (MEDLINE, Embase, PubMed, Cochrane Central, and Scopus) between January 2007 and September 2022. The key search derived from the medical subject heading terms were the following: Tubulinopathies, or *TUBA1A*-related encephalopathies; or *TUBA1A*-related disorders; or *TUBAIA* and Cerebral Malformation, or *TUBAIA* and cerebellar malformations. After examining 58 articles related to this topic and removing duplicate records, 32 studies were included,

## Results

The 32 selected articles on TUBAIA-tubulinopathies were as follows: 12 were related to genetic results, 13 to systemic clinical manifestations, and seven were case reports. Pathogenic variants in *TUBA1A* were associated with cortical and subcortical malformations and with severe neurological complications. The mechanisms of *TUBA1A* mutations in causing these severe cerebral anomalies remain unclear.

## Discussion

Pathogenetic variants of TUBAIA, as observed by Fallet-Bianco et al. (15), affect several cerebral cortical and subcortical structures with various and severe clinical manifestations. Clinical features related to subcortical and cerebral TUBAIA-tubulinopathy are reported.

### *TUBA1A*-tubulinopathy and subcortical malformations

In a large-exome sequencing study in 282 individuals from 100 families with Dandy–Walker malformation (DWM) and cerebellar hypoplasia (CBLH), Aldinger et al. (19) obtained a molecular diagnosis in 36 families with a significantly higher number for CBLH (51%) than for DWM (16%). Among the 41 variants, only 27 neurodevelopmental disorder–associated genes were found, thus showing that CBLH and DWM are more often features of monogenic neurodevelopmental disorders. The authors (19) reported a newborn with DWM and *TUBA1A* mutation. Brain MRI showed small cerebellar hemispheres, an unpaired caudal lobule (“the Dandy–Walker tail”), and a single periventricular nodular heterotopia. Hebebrand et al. (5) identified three patients with heterozygous *de novo* missense variants in *TUBA1A* and reviewed the cases reported in the literature. About 166 patients were enrolled with 146 born and 20 fetuses and 107 cases showing available clinical information. The most commonly reported features were developmental delay (98%), anomalies of the corpus callosum (96%), microcephaly (76%), and lissencephaly (agyria–pachygyria) (70%). The authors (5) identified a total of 121 specific variants, including 15 recurrent and three new cases of DWM with a detailed clinical history and presentation of the associated dysmorphic features (cases description is reported in the Supplementary material).

### *TUBA1A*-tubulinopathy and related cerebral malformations

Keays et al. (1) first reported two patients with *TUBA3* mutations (the human homolog of *TUBA1A*) both presenting with lissencephaly, pachygyria, and hippocampus anomalies. Among 95 sporadic patients with non-syndromic bilateral PMG, including 54 patients with perisylvian PMG and 30 with PMG and additional brain abnormalities, Poirier et al. (9) identified three unrelated patients with mutations in *TUBA1A* representing 3.1% of the PMG group and 10% of PMGs with complex cerebral malformations. The last group included patients with bilateral perisylvian asymmetrical shapes, PMG with dysmorphic basal ganglia, cerebellar vermian dysplasia, and pontine hypoplasia. The authors (9) suggested that in addition to PMG, additional brain abnormalities such as dysmorphic basal ganglia, hypoplastic pons, and cerebellar dysplasia are relevant features for PMG *TUBA1A*-related diagnosis. The authors (9) concluded that patients with *TUBA1A* mutations share not only cortical dysgenesis but also cerebellar, hippocampal, corpus callosum, and brainstem abnormalities. Four fetuses with *TUBA1A* mutations and a prenatal diagnosis of major cerebral malformations with termination of pregnancy were reported by Fallet-Bianco et al. (15). The pathologic studies of the fetuses at 23, 25, 26, and 35 gestational weeks showed a spectrum of abnormalities, which encompassed five brain structures: the neocortex, hippocampus, corpus callosum, cerebellum, and brainstem. Other abnormalities involved the basal ganglia, olfactory bulbs, and germinal zones. Abnormal cortical and hippocampal lamination and heterotopic neurons in the cortex, cerebellum, and brainstem were found during the microscopic examination (15). Six patients with *TUBA1A* mutations were reported by Bahi-Buisson et al. (4). The authors showed that the *TUBA1A*-related lissencephaly spectrum ranges from perisylvian pachygyria in the less severe form to posteriorly predominant pachygyria in the most severe, associated with dysgenesis of the anterior limb of the internal capsule and mild to severe cerebellar hypoplasia. Mutation analysis in the *TUBA1A* gene was performed by Morris-Rosendahl et al. (20) in 46 patients with classical lissencephaly. They identified three novel pathogenic variants and one recurrent mutation in five patients with variable patterns of lissencephaly on the brain MRI. Congenital microcephaly was found in four of the five patients, and all showed dysgenesis of the corpus callosum, cerebellar hypoplasia, and variable cortical malformations, including subtle cortical band heterotopia and absence or hypoplasia of the anterior limb of the internal capsule. The authors maintained that the frequency of mutation in *TUBA1A* affects approximately 4% of the patients with lissencephaly. Kumar et al. (8) conducted a study of *TUBA1A* mutations in 125 patients with cortical dysgenesis including 72 patients with classical LIS, 22 with subcortical band heterotopia (SBH), 29 with LIS and cerebellar hypoplasia (LCH), and two with LIS and agenesis of corpus callosum (ACC). The results obtained consisted of missense mutations in five out of 72 patients with classic LIS (7%) and 10 missense mutations in 29 patients with LCH (32%). *TUBA1A* mutation was also found in one child with ACC and CBLH without LIS. The authors (8) summarized the brain MRI features of 17 patients with *TUBA1A* mutation in five distinct groups according to the observed variants. Three patients with *TUBA1A* mutations were reported by Jansen et al. (21) with classic LIS in a boy and polymicrogyria in two sisters whose mother showed somatic mosaicism, thus showing familial recurrence of the mutation. A single case in a 14-month-old girl with *TUBA1A* mutation and classical lissencephaly was reported by Sohal et al. (14). Marked ventricular dilatation with thin cortex, poorly differentiated basal ganglia, agenesis of corpus callosum, and cerebellar hypoplasia with preserved vermis were described by Okumura et al. (22) in a child with *TUBA1A* mutation and cerebellar hypoplasia. An extensive neuropathologic analysis showed a lacking lamination in the cerebral cortex, absent corpus callosum without Probst bundle, blurred demarcation among the striatum internal capsule and globus pallidus, irregular running of myelinated fibers, cerebellar hypoplasia with irregular undulation in the dentate nucleus and inferior olivary nucleus, absent olfactory bulbs and tracts, and pyramidal tract hypoplasia. Among 106 patients selected as having complex cortical malformations, Bahi-Buisson et al. (23) identified 45 (42.5%) patients presenting with mutations in *TUBA1A*, 18 in *TUBB2B*, (16.9%), 11 in *TUBB3* (10.4%), three in *TUBB5* (2.8%), and three in *TUBG1* (2.8%). Five cortical malformation syndromes were distinguished by the authors (23) in a systemic revision of the data collected by neuroimaging and neuropathologic analysis in patients affected by *TUBA1A* and other tubulinopathies: microlissencephaly (*n* = 12), lissencephaly (*n* = 19), central pachygyria and polymicrogyria-like cortical dysplasia (*n* = 24), generalized polymicrogyria-like cortical dysplasia (*n* = 6), and simplified gyral pattern with area of focal polymicrogyria (*n* = 19).

Dysmorphic basal ganglia were reported in 75% of cases and central pachygyria, polymicrogyria-like cortical dysplasia, and simplified gyral malformations syndrome in 100% of the cases. Moreover, the authors (23) highlight a high prevalence of corpus callosum agenesis (32/80; 40%) and mild to severe cerebellar hypoplasia and dysplasia (63/80; 78.7%). Fallet-Bianco et al. (24) selected 19 cases of *TUBA1A* mutations in a cohort of 60 fetal cases. The authors refer that all the cases of lissencephaly with cerebellar hypoplasia showed distinct *TUBA1A* mutations, while those with classical lissencephaly harbored recurrent mutations in *TUBA1A* (three cases) or *TUBB2B* (one case). The authors (24) concluded that fetal *TUBA1A*-tubulinopathies most often present with microlissencephaly or classical lissencephaly with corpus callosum agenesis, but polymicrogyria may also occur in contrast to *TUBB2B* mutations in which either polymicrogyria or microlissencephaly may be found. *TUBA1A* mutations affect almost exclusively the central nervous system (CNS), but congenital malformations may involve other body organs as reported by Hikita et al. (25) in a 6-year-old girl who presented with lissencephaly, microcephaly, and early-onset epileptic seizures in addition to Hirschsprung disease and inappropriate antidiuretic hormone secretion (SIADH). In a cohort of 156 patients with malformations of cortical development of unknown origin, 79 patients were selected and submitted to a genetic analysis of the *TUBA1A*, *TUBB2B*, and *TUBB3* (26). Two novel heterozygous mutations were found: a *TUBA1A* mutation in a 5-year-old female with microcephaly, severe intellectual disability, and absence of language and a *TUBB2B* in a 31-year-old female with microcephaly, spastic tetraparesis, severe intellectual disability, and scoliosis. The patient with *TUBA1A* mutations in the brain MRI showed pachygyria associated with a diffuse subcortical band heterotopia that spared the fronto-basal regions only; the heads of caudate nuclei and the putamen were fused; corpus callosum was thin with anterior commissure absent; the hippocampi showed a simplified pattern. Abnormal transition between the medulla and a-flattened pons, thickened mesencephalon, and a thinned pontomesencephalic junction were also present. The cerebellar vermis was mildly hypoplastic. Based on the results of the genetic analysis obtained in the patients, the authors (26) concluded that the frequent observation of hypoplastic and disorganized white matter tracts led to suggest that in addition to defects in neuronal migration, disruption of axon growth and guidance represent a peculiar feature of the disorders linked to tubulinopathy. Shimojima et al. (27) reported a single case of a Japanese baby girl with microcephaly, lissencephaly with cerebellar hypoplasia, and corpus callosum hypoplasia. Genetic analysis disclosed a region anomaly that encodes the N-terminal domain of TUBA1, a region uncommonly involved. The brain MRI showed the presence of colpocephaly, lateral ventricle dilatation, and simplified gyral pattern. There was also hypoplasia of the corpus callosum and the cerebellar vermis. A *TUBA1A* variant was reported by Bosemani et al. (28) in a 22-month-old girl with developmental delay. Brain MRI showed vermian hypoplasia with abnormally increased rostrocaudal length of the medulla and midbrain involving the pons, and loss of the normal flat dorsal surface of the brainstem. In a study on patients with hindbrain imaging abnormalities, Oegema et al. (29) identified 10 patients. In seven of 10 patients (78%), targeted sequencing revealed mutations in three different tubulin genes (*TUBA1A*, *TUBB2B*, and *TUBB3*) occurring *de novo* or inherited from a mosaic parent. Brain MRI revealed cerebellar dysplasia combined with basal ganglia dysplasia in all of the cases (100%) and brainstem dysplasia in almost all (80%), and only irregular patterns of cortical gyri and sulci. With regard to the two patients with *TUBA1A* mutations, they showed microcephaly, severe developmental delay, and epilepsy. The MRI features in both cases showed diffuse irregular gyration and sulcation of cortex, vermis hypoplasia, asymmetry of pons, medulla dysplasia (in one case), partial agenesis of corpus callosum, basal ganglia hypoplasia, enlarged ventricle, and cranial nerve hypoplasia. A 9-year-old girl affected by epileptic seizures, developmental delay, hypotonia, and microcephaly was reported by Mencarelli et al. (30). The girl showed mild facial dysmorphism including bulbous nasal tip, large mouth, edema of the hands and feet with camptodactyly, bilateral thelarche, hypoplasia of the labia minora, and poor visual and social interaction. The girl had a *de novo* heterozygous mutation in *TUBA1A* gene. Brain MRI showed complex cerebral malformations with mild asymmetry and dilatation of lateral ventricles and reduced white matter. Cortical dysgenesis with dysmorphic frontal lobes, simplified gyral pattern, and poor development of the Sylvian fissure were also present. The corpus callosum was thin and basal ganglia were hypoplastic. Right caudate nucleus and right lenticular nucleus were dysmorphic. Cerebellar vermis and pons hypoplasia were also reported. A study was conducted by Romaniello et al. (31) on 28 patients harboring 23 heterozygous pathogenic variants in tubulin genes *TUBA1A* (*n* = 10), *TUBB2B* (*n* = 8), and *TUBB3* (*n* = 5). Neuroimaging patterns of patients showed cerebellar dysplasia and other posterior fossa morphological anomalies. Cerebellar anomalies were found in 24/28 patients (86%): cerebellar dysplasia in 19/28 (68%) including cortical cerebellar dysplasia (18/28), either involving only the cerebellar hemispheres (12/28) or associated with vermis dysplasia (6/28). Cortical cerebellar dysplasia was found only in the right hemisphere in 13/18 including four *TUNN2B*, four *TUBB3*, and five *TUBA1A* mutations. In contrast to the remaining five *TUBA1A* patients, cortical cerebellar dysplasia was located only in the left hemispheres or in both. The authors refer that the cerebellar involvement in tubulinopathies shows peculiar aspects that may be labeled as “tubulin-related cerebellar dysplasia” (31). Epilepsy is common in patients with *TUBA1A*. In a cohort of 15 patients and 75 from published studies, Romaniello et al. (32) reported epileptic disorders in 60% of the patients affected by *TUBA1A* mutations, 74% by *TUBB2B*, and 25% by *TUBB3*. In these patients, brain MRIs were consistent with the extensive brain malformations involving subcortical and midline structures (32).

## Conclusions

Genotype–phenotype characterization of structural brain abnormalities in patients with *TUBA1A*-tubulinopathies is limited by the large interstudy variability in reporting the clinical features of this disorder. Indeed, previous reports showed the various types of cerebral malformations related to this genetic disorder and their association with severe neurological and systemic manifestations.

Pathogenetic variants in *TUBA1A* are associated with a broad spectrum of cortical and subcortical brain malformations. Major cortical abnormalities include “classical” lissencephaly, lissencephaly with agenesis/or dysgenesis of the corpus callosum, lissencephaly with cerebellar hypoplasia, polymicrogyria, and mild to moderate dysgyria. Dysmorphic basal ganglia, thalami, and corpus callosum may also be involved as well as cerebellar vermis and cerebellar cortical dysplasia. Brainstem malformations may present with usually asymmetric hypoplasia. Clinical manifestations are variable and usually severe, including motor and cognitive impairment. Developmental delay, intellectual disability, cerebral palsy, and seizures often unresponsive to treatment are widely reported. Undistinctive facial dysmorphism, microcephaly, other associated malformations, and cerebral MRI anomalies may support genetic testing for the suspect of *TUBA1A*-tubulopathy. Supportive treatments including physical, occupational, speech therapy, and anticonvulsant treatment are necessary although the prognosis remains quite poor, especially in most severe cases (see Table 1).

**Table 1:** Brain MRI. Main cerebral malformations in patients with *TUBA1A*-tubulinopathy.

Authors	Patients with *TUBA1A*-tubulinopathy	Brain MRI—cerebral malformations
Keays et al. (1)	2	Lissencephaly, pachygyria, hippocampus anomalies
Poirier et al. (9)	3	Bilateral perisylvian asymmetrical shapes, PMG with dysmorphic basal ganglia, cerebellar vermian dysplasia, pontine hypoplasia
Fallet-Bianco et al. (15)	4 fetuses	Pathologic study—five brain structures involved: neocortex, hippocampus, corpus callosum, cerebellum, brainstem
Bahi-Buisson et al. (4)	6/106 complex cortical malformations	Perisylvian pachygyria (less severe form): posteriorly predominant pachygyria (most severe form), internal capsule dysgenesis, mild to severe cerebellar hypoplasia
Morris-Rosendahl et al. (20)	5/46 lissencephaly	Variable pattern of lissencephaly, dysgenesis of corpus callosum, cerebellar hypoplasia, variable cortical malformations
Kumar et al. (8)	125 cortical dysgeneses	72 classical lissencephaly 22 subcortical band heterotopia 29 lissencephaly + cerebellar hypoplasia 2 lissencephaly + corpus callosum agenesis
Jansen et al. (21)	3/25 cortical development malformations	1 lissencephaly 2 polymicrogyria
Sohal et al. (14)	1	Lissencephaly
Okumura et al. (22)	1	Ventricular dilatation with thin cortex, poorly differentiated basal ganglia, corpus callosum agenesis, cerebellar hypoplasia with preserved vermis
Fallet-Bianco et al. (24)	19/60 fetuses	Pathologic study: lissencephaly and cerebellar hypoplasia distinct features in *TUBA1A* mutations
Hikita et al. (25)	1	Lissencephaly, microcephaly, Hirschsprung disease
Romaniello et al. (26)	79/156 diagnosis of cortical malformations	Pachygyria, heads of caudate nuclei and the putamen fused, thin corpus callosum, hippocampi simplified pattern, cerebellar vermis mildly hypoplastic
Shimojima et al. (27)	1	Colpocephaly and lateral ventricle dilatation, simplified gyral pattern, hypoplasia corpus callosum and cerebellar vermis
Bosemani et al. (28)	1	Vermian hypoplasia, abnormally increased rostrocaudal medulla and midbrain, loss of flat dorsal surface of the brainstem
Oegema et al. (29)	2/10 hindbrain imaging abnormalities	Vermis hypoplasia, pons asymmetry, partial corpus callosum asymmetry, diffuse irregular gyration and cortex sulcation
Mencarelli et al. (30)	1	Mild asymmetry and dilatation of the lateral ventricles, cortical dysgenesis with dysmorphic frontal lobes-simplified gyral pattern, cerebellar vermis and pons hypotrophy
Romaniello et al. (31)	5/28 harboring tubulin gene variants	Cortical cerebellar dysplasia
Romaniello et al. (32)	48/90 *TUBA1A* epileptic patients	Extensive brain malformations involving subcortical and midline structures

## Data Availability

The original contributions presented in the study are included in the article/supplementary material, further inquiries can be directed to the corresponding author/s.
