# Supplementary material for: Case report: Structural brain abnormalities in TUBA1A-tubulinopathies: a narrative review
Source: Front Pediatr. 2023 Sep 8;11:1210272. doi: 10.3389/fped.2023.1210272 (PMC10515619; doi:10.3389/fped.2023.1210272)
Supplement: Supplementary file 1 [file Datasheet1.pdf]

Supplementary file: detailed description of the case reported by Hebebrand et al (5)

Case 1, a 13-year and 7 months-old boy manifested with developmental delay, seizures, and muscular hypotonia. Brain MRI revealed coarsened cerebral gyri, hypoplasia of corpus callosum, cerebellar vermis hypoplasia, ventricular dilatation, and narrowed white matter. The patient showed minor craniofacial dysmorphism consisting of a flat forehead, low set ears, epicanthic fold, upward slanting palpebral fissures, narrow nasal bridge, broad nasal tip, short philtrum, and everted lower lip. In addition, clinodactyly, small forefoot and sandal gap were noticed. The second case, a 11 years and 6 months old boy showed global developmental delay. Brain MRI revealed mild frontal cortical anomalies, hypoplasia of corpus callosum, basal ganglia dysgenesis, ventricular dilatation, accentuated lamina quadrigemina, and retrocerebellar arachnoid cyst. Minor craniofacial dysmorphism consisted of high forehead, large earlobes, epicanthic fold, hypertelorism, jaw deformity, cupid bow-shaped upper lip, with open mouth appearance, high arched palate and gap between the upper incisors. Minor features involved the thumbs distally located, pointed fingers, hallux valgus and sandal gap. The third case a 9 years and 3 months old girl featuring muscular hypotonia, nystagmus, developmental delay, and epilepsy. Cerebral MRI revealed a Dandy-Walker variant syndrome with cerebellar vermis hypoplasia, agenesis of the corpus callosum, ventricular dilatation, dysgenesis of basal ganglia, and unilateral optic nerve. Minor facial dysmorphism included large ears, hypertelorism, broad flat nasal bridge, high arched palate, wide-spaced teeth, thin lips, short neck, smooth philtrum and palmar simian crease. Additional features were proximally located thumbs, pointed fingers, brachymesophalangia finger V, and clinodactyly finger V (5).
